# The Impact of the COVID-19 Pandemic on the Working Lives and Retirement Timing of Older Nurses in Ireland

**DOI:** 10.3390/ijerph181910060

**Published:** 2021-09-24

**Authors:** Áine Ní Léime, Margaret O’Neill

**Affiliations:** Irish Centre for Social Gerontology, ILAS Building, National University of Ireland Galway, H91C7DK Galway, Ireland; margaret.oneill@nuigalway.ie

**Keywords:** COVID-19, older nurses, retirement, health, wellbeing, healthcare, Ireland, gender

## Abstract

COVID-19 profoundly affected Irish citizens. The effects have been especially pronounced for nurses in front-line, clinical and management roles. This article discusses the national and employer policy context relevant to nurses in Ireland. There have been staff and bed shortages in public hospitals since austerity policies were introduced following the global financial crisis. Government measures responding to the pandemic include initial ‘cocooning’ of older citizens, travel restrictions, changed working conditions and restricted availability of childcare. This article draws on interviews with 25 older nurses in 2021, sixteen women and nine men, aged 49 or over in Ireland. It explores older nurses’ experiences of COVID-19 and asks what are the implications for their working conditions and retirement timing intentions. A gendered political economy of ageing approach and thematic analysis reveals that while some nurses responded positively to the pandemic, some experienced adverse health impacts, stress and exhaustion; some reported a fear of contracting COVID-19 and of infecting their families; several women nurses decided to retire earlier due to COVID-19. The implications of the findings for employer and government policy and for research are discussed.

## 1. Introduction

The COVID-19 pandemic, declared by the World Health Organisation (WHO) in March 2020, has given rise to an unprecedented global health crisis. In Ireland, which has a population of close to five million, 5179 people have died from COVID-19 since the start of the pandemic, at the time of writing [1]. The Irish government has introduced varying levels of lockdown to contain the pandemic. Along with other policy measures, including the closure of schools and childcare facilities and the direction that all adults aged 70 and over should ‘cocoon’ at home to avoid contact with other people [2], this has had a strong impact on older workers, including nurses in Ireland. The situation has challenged healthcare systems across the world and has seen dramatic changes in ways of delivering healthcare. There is limited international research on the impacts of the pandemic for the working conditions and lives of older nurses and no research on this topic in Ireland. There is a pre-existing problem in Ireland in relation to the retention of nurses [3]. Understanding the experiences of older nurses is important to facilitate them to work for longer if they wish. This exploratory study, based on interviews with 25 nurses, provides valuable insights to help inform organizational policy and will contribute new insights to the literature on the impact of catastrophes on the timing of retirement. The need for research on retirement in the face of uncertainty has recently been highlighted in the context of the promotion of extended working lives in OECD countries [4].

Addressing this gap in existing knowledge, this article uses a gendered political economy of ageing framework to understand the impact of COVID-19 on the work-life experiences and retirement decisions of nurses [5]. This approach understands the meaning and experience of ageing in the context of broader socioeconomic determinants and public policy interventions [6]. It considers the experience of ageing in work as gendered and shaped not only by subjective experience and agency but also by broader structures of legislation and policy, organisation and (family) caring responsibilities [7]. This exploratory study asks what are the main impacts of the pandemic for the working lives of older nurses in Ireland, including their own and their families’ health and wellbeing, and connectedly, how does the pandemic affect their decisions regarding their timing of their retirement? Considering nurses’ critical role on the front line of healthcare during the pandemic, understanding these issues will help inform future directions for employment policies for nurses in Ireland in a health system that was already under-resourced prior to the pandemic and at a time when the government is encouraging workers to extend their working lives [8,9,10].

In Ireland, emergency public health measures included restructuring, changes in work team allocations, very restricted or no patient visiting and a vast amount of new information and protocols for staff to follow [9]. Healthcare workers, particularly nurses, were faced with unprecedented and ongoing challenges. There is limited research on the lived experiences of nurses of the pandemic internationally, relatively little on how nurses in Ireland respond to the situation, and no studies focusing specifically on older nurses. Yet, over 60% of staff nurses in Ireland are aged over 40 while nearly a third (28.5%) are aged over 50 [11]. Understanding their experiences is important to ensure that appropriate policy provisions and employer supports are in place to facilitate retention of nurses and provide a high standard of healthcare, particularly during this critical time. Nurses play a central role in tackling public health emergencies; they are responsible for direct patient care and for mitigating the risk of disease infection and are at high risk of personal exposure [10].

The article contributes to the sociology of ageing and employment, building the empirical picture of the impact of global catastrophes on nurses’ working conditions and experiences and on their retirement decision making. The findings provide critical information that can inform health authorities to enable them to improve working conditions for nurses and positively impact on the quality of care provision. By focusing on the impact of retirement decisions, the article offers insights into the factors or combinations of factors that may nudge older nurses into leaving the workforce prematurely. Since the research took place after the pandemic has been ongoing for a year, it offers insights into the impacts of a long-running pandemic for the nursing workforce, adding to the small but growing body of qualitative research internationally on this issue. This article reviews the literature relevant to nurses’ experiences of the pandemic internationally and in Ireland, outlines the gendered political economy of ageing approach, which provides a broad framework for the analysis, gives an overview of methods and discusses the findings and their implications for policy and for future research. The findings provide insights into the ways in which working through the pandemic has affected the views of older Irish nurses on their work and retirement decisions.

### 1.1. Economic Conditions and Nursing in Ireland

In Ireland, the pandemic has highlighted significant under-investment over many years in the hospital and long-term care system. The Irish health system experienced severe cutbacks due to financial austerity measures introduced in response to the global financial crisis [12]. Nurses employed in the public sector by the Health Service Executive (HSE) have been particularly affected by cost-control strategies, with an increasing workload and higher patient ratios [3]. Nursing in Ireland is highly feminised; in 2019, 90.8% were female [13]. However, the proportions vary across sectors—35% of registered psychiatric nurses are men in 2017, while only 7% of registered general nurses are men [14]. The HSE nursing turnover rate was 7.3% in 2019 (up from 2.9% in 2014) [3]. High turnover rates and difficulties with staff retention are attributed to ‘poor workforce planning, high occupational stress, low job satisfaction and poor wellbeing’ leading to staff shortages [3]. An international review of previous pandemic situations demonstrates that many healthcare workers are willing to accept occupational risks; however, nurses, women and younger workers are more likely to perceive the risks to be too high [10]. As previous research shows that some nurses choose to leave their jobs during pandemics, this impacts on the workforce and the ability of health systems to deliver care [10]. Research on Irish nurses’ perceptions of preparedness for an influenza pandemic reveals a commitment to care, but also concerns about an increased risk of personal infection, their families’ health and additional work pressures and stress [15]. However, there is little research on Irish nurses’ direct experiences of working through pandemics and none focusing on the level of occupational risk older nurses are willing to accept. Understanding the factors that can inform Irish nurses’ decisions to stay or leave in such times is important to inform healthcare system strategies and employment policies.

### 1.2. Occupational Health Risks for Nurses during COVID-19

Nurses in Ireland and internationally are at increased occupational risk of contracting COVID-19. In June 2020, nearly one in three COVID-19 cases in Ireland involved healthcare workers, and one in ten were nurses or midwives. More than two thirds of these workers contracted the virus at work [16]. While many workers stayed at home to minimise the transmission of the disease, nurses in hospitals and community settings continued to work in high-risk areas. Both internationally and in Ireland, there were initially shortages of personal protective equipment (PPE) [2,17,18]. In Ireland, to preserve supply, PPE was initially limited to staff working directly with diagnosed COVID-19 patients [19]. This adversely affected the working conditions and health of nurses. Nurses have been significantly affected by pressures arising from the pandemic. Interviews with US ICU nurses identified challenges in adapting to safety and care procedure changes, working with new co-workers and teams, a lack of information about the virus and leaving their home units [20]. Common COVID-19 health impacts on healthcare workers internationally include fever, cough, weakness, skin damage due to use of PPE and high levels of depression, anxiety, insomnia and distress [21]. Factors correlating with increased risk of mental health problems were exposure to COVID-19 patients, being a woman, worry about being infected and about family members being infected [21,22,23,24]. For some, the need to protect their family from infection contributes significantly to the risks and burdens of working during the pandemic [25,26,27]. International qualitative studies show that pandemic fatigue appears to arise when early negative emotions become reactivated due to the ongoing pandemic [27]; however, positive emotions, including resilience and growth under pressure, have also been identified [10,28,29]. There is a need for qualitative research to investigate the effect of these impacts in Ireland and the responses of Irish nurses in terms of remaining in/exiting from the workforce.

### 1.3. Organisational Policies and Health Challenges for Nurses in Ireland during COVID-19

When the pandemic struck, healthcare workers in Ireland had to adapt to the stresses of responding to sudden and significant changes to their daily work practices [9]. Prior to COVID-19, there were no identifiable policies in Ireland on the delivery and staffing of healthcare services during pandemics [19]. During the first wave, policies for the reorganisation and redeployment of staff were prioritised. In hospital settings, staff were redeployed to areas of high clinical need. In community settings, they were redeployed to COVID-19 testing and contact tracing. An Irish Nurses and Midwives Organisation (INMO) survey on the health concerns of the impact of COVID-19 for nurses in Ireland found high levels of negative psychological impact, as well as mental exhaustion while off duty, and concern about personal health and the risk of spreading the infection to family and housemates. Significantly, 61% considered leaving the profession [3]. Beyond the workplace, COVID-19 restrictions compounded these challenges due to creche closures, lack of day services for older people and limited access to normal coping strategies such as opportunities for social connection and leisure activities [25]. Prior to the pandemic, Irish healthcare workers were identified in the European Working Conditions Survey to report high rates of burnout, placing them at risk of occupational stress post-COVID-19 [25]. The INMO study suggests that during COVID-19, nurse staff shortages have been very high and that it is critical to investigate the risks posed by the pandemic to nurses [3]. A small body of work comprising qualitative, reflective and observational cohort studies on the experiences of healthcare workers, including nurses, in Ireland, has found that the most challenging aspects of the pandemic have been limited resources and understaffing [3,30], adapting models of care [31] and psychological distress and occupational stress [9,32]. Our research adds to this small but growing body of knowledge by considering how gender or age implications may influence the responses of nurses working through the COVID-19 pandemic in Ireland in the context of these policies and health challenges and the impact on nurses’ retirement decisions.

### 1.4. Gendered Caring Norms and COVID-19 Policies in Ireland

Historically, women were regarded as the primary carers for family members in need of care in Ireland and had low labour force participation rates [33]. Women are still regarded as primary carers both for children and for older family members, although to a somewhat lesser extent in recent years. A recent report identified the peak age for women to provide unpaid care for older or other family members in need of care as 50–64 [34]. As mentioned previously, many childcare and day care services for older people were closed during the pandemic. We might expect that this situation would place additional pressure on older nurses who help care for grandchildren and their own parents or spouses in addition to working. There is one workplace policy available for nurses in clinical roles, a pilot pre-retirement initiative, that provides for some eligible nurses to work part time for up to five years before retirement and retain full-time pension rights. This should help nurses who wish to combine work with unpaid family caring responsibilities. However, this is available only to nurses in clinical roles. This article provides insights into whether and how caring responsibilities may affect nurses’ decisions to stay on or leave work.

### 1.5. The Impact of COVID-19 on Nurses’ Retirement Timing

The pandemic has challenged nurses in terms of reorienting how they understand and value their role. Qualitative studies internationally have found a conflict between professionalism and fear for personal and family health [35,36]. However, a qualitative review observes that professional commitment tends to support nurses in their dedication on the front line despite the risks and deepens their understanding of their role [27]. For instance, nurses have been shown to centralise care—valuing close connection with patients over a task-based focus [29,35]. In terms of the social perception of the nursing role, international qualitative studies reveal that nurses feel valued by society and consider that the prestige of the profession has been elevated during COVID-19 [27]. However, in an Irish and UK interview-based study of frontline workers, participants provided mixed responses to overt gestures of appreciation, as while many appreciated them, some felt that it put them under additional pressure, and some perceived a lessening sense of social solidarity [32]. Health, family considerations and job satisfaction represent important factors in nurses’ retirement decision making; there are significant associations between gender, home situation and the intention to retire early [37]. The current situation gives rise to the need to explore how a pandemic-induced evaluation of the risks and rewards of their role may affect nurses’ retirement decisions in Ireland. Such decisions are already informed by personal and financial (pension) considerations, socioeconomic conditions and national and organisational policies, which are now additionally complicated by COVID-19. Understanding changes in how nurses perceive their professional role, as well how they consider society to view the role, is critical to informing knowledge on how the profession can be developed and the workforce supported during the current crisis. Moreover, this information can inform societies and governments about how to support older workers during the pandemic and beyond.

## 2. Theoretical Framework and Methodology

### 2.1. Theoretical Framework

Using a gendered political economy of ageing approach involves considering how the experiences of older nurses in the workplace in Ireland are shaped by social and structural forces in the economy. Such forces include the impact of gendered divisions of labour, for instance regarding caregiving responsibilities within the family, and organisational practices, such as workplace flexibilities or otherwise, surrounding these commitments. Given that limited retirement options can affect financial security and the question of when to depart from the labour force, these factors influence nurses’ decisions to stay or leave even during a time of crisis such as the current pandemic. The age-related policy of ‘cocooning’ individuals aged 70 and over has also impacted workers in Ireland, for instance those over 70 who wished to continue working or provide support for other workers. More broadly, this approach also takes into account the consequences of policies of austerity that were introduced following the global financial crisis of 2008 and a severe recession in Ireland. Resulting regressive social policies and public spending cuts included cuts in health and social care, leading to embargoes on nurse recruitment. Furthermore, public spending cuts in the health system in recent years have seen reduced staff numbers as well as pay cuts for the nursing workforce, so that a higher workload and reduced pay places them under increasing pressure, with weakened social protection [3,12,19]. This study considers how these dynamics of gender and age, shaped by State and employer policies, and economic market forces, inform the subjective experiences and perceptions of working life for nurses in Ireland during COVID-19.

### 2.2. Research Questions

A qualitative study was undertaken to explore the experiences and views of older nurses (aged 49 and older) in Ireland in the context of working through the COVID-19 pandemic, specifically in relation to the effects on their working and family lives. This exploratory study asks the following research questions: (1) What are the main impacts of the pandemic on the working lives of older nurses in Ireland, including their own and their families’ health and wellbeing? (2) How does the pandemic affect their decisions regarding retirement timing?

### 2.3. Participants and Methods

Twenty-five nurses aged 49 or over participated in interviews about their experience of COVID-19 during the pandemic. The interviews took place between late-January and mid-April 2021—11–13 months into the pandemic. These were follow-up interviews conducted as part of a larger European project, Dynamics of Accumulated Inequalities for Seniors in Employment (DAISIE), where interviews were conducted with 40 nurses—24 women and 16 men—about their work-life history and their views on extended working life. A link to the DAISIE website is in the Funding section below. For the larger project, participants were approached via gatekeepers in the health sector and through snowball sampling. In January 2021, participants from the larger project were contacted by email to ask if they would consider taking part in the current follow-up study. Twenty-five nurses agreed to participate, sixteen women and nine men. All interviews except one were conducted by telephone, with one conducted via Microsoft Teams. Interviews lasted approximately 15 min on average. Interviews were recorded, transcribed verbatim, and data were anonymised. Pseudonyms are used throughout the article. The findings here are based on these re-interviews.

The nurses were asked specifically about their experiences of COVID-19; whether and how it affected their work, their family lives, their health and any caring responsibilities and whether and how the pandemic has affected their perception of their role as a nurse and their decisions around retirement timing. Finally, they were asked whether they would recommend any policies for the government or for employers. Interviews were manually coded and analysed to identify themes. Both authors coded the interviews independently and then discussed and compared their findings until overarching themes emerged.

### 2.4. Profile of Participants

The nurses worked across a range of settings including acute hospitals, community care, community mental health settings and centres for intellectual disability. Most nurses were employed by the Health Services Executive (HSE)—the Irish national health service—and were serving in a wide range of grades from Staff Nurses to Clinical Nurse Managers to Assistant Directors of Nursing (ADON) (see Table 1). The men were fairly evenly distributed across three age groups, with a somewhat higher concentration of women at the middle (55–59) and higher (60–64) age groups (see Table 2). Approximately two thirds of the participants were married or in a long-term relationship, while under one third were divorced, and two were single (see Table 3). Seven of the participants—five women and two men—had caring responsibilities for relatives (see Table 4).

### 2.5. Analytic Approach

Thematic analysis was chosen to explore shared meaning-based patterns across the data on how the nurses’ work-life experiences have been informed by the COVID-19 pandemic, as well as exploring the implications of working through the pandemic for their retirement decision making [38]. A reflexive approach following Braun, Clarke, Hayfield and Terry [38] provided for capturing meaning across varied contexts of work and home and for considering the essence of the nurses’ experiences, with themes emerging from the researchers’ analysis. This approach complemented the political economy of ageing framework when it came to considering the arising themes in the context of broader socioeconomic determinants.

## 3. Results

The findings provide insights into the ways in which the pandemic affects the working lives of older Irish nurses, specifically the ways it impacts on their health, how it influences their own and the publics’ perceptions of their role as nurses and the impact, if any, on their retirement timing.

### 3.1. Impact on Working Lives

Almost all of the nurses said that their work and working conditions were affected (typically adversely) by the pandemic and some invoked rhetoric more typically associated with war or sport to describe their experiences. A small number reported having a positive perspective on their experience, and again, some deployed military rhetoric in their descriptions. Several nurses were redeployed and/or had to take on new roles in addition to their current role; many were exhausted from a combination of the precautions they needed to take, the fear of contracting COVID-19 themselves and/or of infecting their vulnerable family members and the fact that many of the settings in which they worked were already short staffed prior to COVID-19. Their tiredness was often exacerbated by the fact that colleagues sometimes had to absent themselves from work either because they had COVID-19 or had a false positive diagnosis, with the result that some of the nurses had not been able to take annual leave for over a year in order to cover for their colleagues.

All the nurses found that their role had changed in some way due to the pandemic. For some, these changes were relatively minor—for example, nurse counsellors having appointments with clients online or managers having online meetings and/or working from home or no longer visiting wards. Some nurse managers reported that their staff were redeployed, particularly at the start of the pandemic, and that they had to take on additional roles and/or train staff for their redeployment roles.

Some were more seriously impacted by the pandemic. One nurse was redeployed to train staff for new roles. As a result of this, she contracted COVID-19 in the early weeks of the pandemic when staff were officially directed not to wear personal protective equipment (PPE), as at that point, PPE supplies were scarce and were given only to those working directly with COVID-19-positive patients, and this exposed nurses to the virus. When asked by the interviewer if she thought she had picked up COVID-19 in the hospital, this nurse explains how she became infected with the virus:

‘Oh I definitely did... it was the time where at the end of March... it was the time we had COVID in every ward and we had no masks. So if you were walking along the corridor, we were asked why we were in masks, like if you weren’t looking after patients, so... they were really looking after the equipment. And I didn’t feel they were looking after the staff’.

(Eileen, Clinical Nurse Manager)

This lack of sufficient PPE and direction not to wear masks also happened in other hospitals in the early days of the pandemic. Another nurse points out that some types of nursing require prolonged close proximity to clients, putting nurses at risk of contracting the virus:

‘But in a caring role in a hospital or in midwifery you can’t step away from somebody. You know, you can’t mind somebody from a distance. And then we didn’t have masks or anything else’.

(Susan, Assistant Director of Nursing)

Several nurses (four women and one man) were redeployed. One describes her experience of being redeployed to a nursing home that had a COVID-19 outbreak and she found it to be extremely demanding, upsetting (dealing with large numbers of dying patients) and exhausting:

‘But on the ground floor, I think there would have been 52 patients, I think we’d 48 that night, 14 were COVID positive and 11 were dying. So that was the beginning of COVID for me. So by the end of April, beginning of May, I was kind of completely burnt out from it, to be honest’.

(Martina, Assistant Director of Nursing)

The nurses report a range of (mainly adverse) impacts on their work with a number of elements combining to make it difficult and stressful to provide care. The requirement to wear personal protective equipment (PPE), which was introduced later, as well as adding to their work-load, makes communication with all patients more difficult. This can be particularly problematic for patients with cognitive impairment or those at the end of life. Some nurses pointed out that chronic staff shortages prior to the pandemic meant that many units were already very stretched and the pandemic was just the last straw. A nurse in an Emergency Department described the situation as follows:

‘But it’s really hard, since 2014, Emergency Departments [such as where I work] have been grossly understaffed, and, you know, year on year, that’s got worse. So, I don’t necessarily think COVID has impacted any more significantly, but it may have been the straw that you know the one that’s going to break the camel’s back because of what’s happened before’.

(Paul, Clinical Nurse Manager)

Another nurse pointed out that the requirement for nurses who have or are suspected of having the virus to isolate at home, together with redeployment of others, makes it even more difficult for their colleagues to maintain services:

‘And of course, there has been an impact. I mean, the hospital is on its knees in terms of... nursing staff now that are pregnant are off and rightly so because of, you know, the risk to their babies and risk to their health. So we’re working on a very negative balance, in terms of providing services... five whole time equivalents out of nine were redeployed. Down to less than half my ordinary [level of staffing] and then one of them was sick... It involved an awful lot of extra work and extra pressure’.

(Marie, Assistant Director of Nursing)

Fear of redeployment also induced anxiety in nurses. Two community-based nurses were told that they could be redeployed at any time to work in nursing homes where there were COVID-19 outbreaks, where they were likely to be exposed to COVID-19. This prospect caused anxiety to these nurses as they felt that they were ill equipped for these roles. This quote illustrates the fear that redeployment engendered:

‘When the manager comes along and tells you... you might have to go into a nursing home, and there’s COVID there... and I haven’t been in a nursing home in 25 years. So, the idea of going in to look after patients who have COVID in an area that I’m not familiar with, and not competent in... frightened the life out of me, and I felt, I’m gonna be more dangerous to them [the patients] than COVID. Because I don’t know what I’m doing... but the idea that you might be moved... that’s what soldiers felt like when they were told, you know, put your bag on and head on to the next place where you might get shot. It is a bit like that’.

(Brenda, Public Health Nurse)

In relation to redeployment, some nurses said that they felt they did not have sufficient upskilling training. Some nurses found themselves covering both their original role and the redeployed role and found this to be exhausting:

‘But for the first say maybe four or five weeks, I was actually trying to be the palliative care nurse, as well as the support for the students [her original role]. So at some stages in the day, I had to kind of get out of one role and into the other role. And I definitely worked extra hours, staying late in the evenings to provide support for students... so how it impacted me then was when I’d come home in the evening I would be absolutely shattered beyond, you know, completely exhausted, completely, you know, completely drained’.

(Bridget, Clinical Nurse Manager)

It was also pointed out that the long duration of the pandemic, in addition to staff shortages, has led to what some nurses term ‘COVID fatigue’. One nurse who had (successfully) worked hard to keep the virus out of the community nursing unit where she worked highlighted this:

‘People get tired, staff get tired, a lot of us have had to step back from our annual leave... because you needed all hands on deck. And if somebody went off, we had a few incidents where we had false positive tests of staff, and so they had to isolate, because we couldn’t take the risk of them being on the unit. And then you have to step up to the mark and... cover the shifts and that. So you’re doing your own shifts, and then you’re taking on somebody else’s shift and bearing in mind, we do twelve and a half/thirteen hour shifts. There’s a lot of tiredness. There’s fatigue—COVID fatigue, I suppose, that’s the thing now’.

(Denise, Senior Staff Nurse)

By contrast, a small number of nurses indicated that the pandemic impacted positively on their working lives—they felt that they were contributing to tackling an important public health crisis—and that they were rising to the challenge, a view articulated by Edwina:

‘But I suppose when the pandemic came, and we were all pushed into doing things that we wouldn’t usually do, and we took it on because it was a pandemic and we’re all being asked to do a bit to help out, that I suppose I realised then that there was probably more left in me than what I thought I had. And that I wasn’t as afraid of a change as I thought I would be. That changes that came along, although they were hugely challenging and continue to be challenging... that I’m, even though it’s, it’s different, and I’m still very much learning the ropes, it’s been, it’s been good so far. I’m enjoying the change’.

(Edwina, Clinical Nurse Manager)

Another nurse working with patients with dementia felt that the pandemic had a positive impact on the relationship between nurses and family members, because they are now in much closer communication:

‘There has been something quite positive coming out of this pandemic, insofar as people weren’t allowed to visit... but it meant that we became, we increased our communication with families—so the relationship between nurses and family members it’s, it’s moving forward. It’s something which we’re really gonna focus on with or without COVID-19 because family members are more trusting now, they’re more accepting and it just builds up our relationship with them’.

(Gerard, Clinical Nurse Manager)

For some nurses, this helped mitigate the fact that it was more difficult to communicate with the patients with dementia while wearing PPE.

### 3.2. Impact on Health of Nurses and Their Families

Nurses reported a range of health impacts from the pandemic both for themselves and for their families. These included: some who contracted the virus and were more or less severely affected by it; others had passed on the virus to their families or were very anxious that they would do so to vulnerable family members; some felt they were not sufficiently protected in the workplace; many of the nurses reported being extremely tired from the extra precautions required at work and the anxiety caused by dealing with the virus and covering for absent colleagues, especially over a long period of time. Two women and two men nurses contracted COVID-19. Two of the nurses contracted the virus at work during the early months of the pandemic when the official guidance was not to wear face coverings unless directly dealing with COVID-19 patients. One of these nurses was severely affected by the virus, resulting in her being admitted to an Intensive Care Unit (ICU) as a patient in hospital for some time and being out of work for over a year. A year later, she is still badly affected; she is very tired and very anxious about contracting the virus again, having almost died from it the first time:

‘I’m tired. I’m very, very tired. But I suppose I’m working from home... and I will be going back to work whenever I’m able. So there’s a little bit of pressure about going back to work like properly in the hospital, but I’ve sort of been able to... delay that just for a little while. Hopefully, until I get my second vaccine, in May, I’ll be able to put off going back until after, because I’m a bit nervous, obviously. You know, I don’t want to pick up something else. I nearly died the last time, you know the way, so I sort of feel very lucky to be here’.

(Eileen, Clinical Nurse Manager)

Members of her family also became infected with COVID-19. Two of her children still have symptoms. She feels that nurses were sacrificed in the fight against the disease and not adequately protected:

‘And as a nurse, I think, I don’t know are we cared for, you know, I do think we in this pandemic, we were fodder, it’s like a war’.

(Eileen, Clinical Nurse Manager)

Another female nurse also developed COVID-19 but recovered relatively quickly—after six weeks. A male nurse contracted the virus. He was ill for only ten days and recovered quickly and completely. However, he highlights the tiredness that he and other nurses are experiencing due to covering for colleagues absent with the virus. At the time of interview, he (and others) had not been able to take annual leave for over a year:

‘I had COVID last year... actually this week, last year, I caught COVID, and I was only ill for probably about ten days. And I made a quick recovery after that. So I’m just very lucky. Everyone’s just tired, not just me, everyone’s just... you’re doing extra shifts, because there’s not enough staff. And everyone’s, we’re just worn out, I’m knackered’.

(Patrick, Clinical Nurse Manager)

This feeling of exhaustion was echoed by several nurses, especially those working in clinical areas. Many nurses were very concerned about their families’ health and were worried about transmitting the virus to vulnerable family members for whom they provided care and/or support, as exemplified by this quote:

‘I suppose, you know, my mother is elderly, and my sister had been unwell. And I was worried and she was in my bubble. So I was kind of worried about her and my mother. So you know, you are, you’re constantly concerned because of the job you’re in, that you’re going to actually give it to someone else’.

(Sarah, Assistant Director of Nursing)

One nurse said that decisions regarding whether staff should be cocooning/protected were not made in a uniform, equitable way across the health service—he used his own situation as an example, where he was not risk assessed by management although he had underlying conditions, while he was required to assess staff in his area who had similar conditions:

‘I would have had a cardiac history… and I’ve worked through all the pandemic, and I haven’t had any of my senior managers coming to risk assess me working within that environment... but yet, I would have had to risk assess other people in being potentially sick enough or vulnerable enough to need cocooning. And so... what I mean by this is, is the standardisation for all members of staff regardless of whether they’re managers, staff nurses, HCA should be implemented, which it’s not’.

(James, Clinical Nurse Manager)

Several of the nurses say that the pandemic has made them become very mindful of their own health and that they now try to ensure that they get enough rest and exercise, or even plan to leave nursing. Some have taken measures such as sleeping separately from their spouses so that there would be someone to look after their children in the event of either of them contracting the virus.

### 3.3. Impact on Retirement Timing

Research participants were asked whether the experience of working through the pandemic had affected their decision regarding retirement timing. There was a clear gender divide. Most of the men said the pandemic did not affect their decision. The following quote is typical:

‘COVID hasn’t influenced that or made me make any decision to get out sooner rather than later. You know, it hasn’t I don’t think’.

(Michael, Clinical Nurse Manager)

Some of the male nurses say that they had started nursing relatively late and the need to continue working for as long as possible to maximise their pension and their financial circumstances was (still) the main influence on their retirement timing. One man says the pandemic has affected his retirement timing decision—he would not want to be retired with nothing to do so it would possibly make him consider working for longer.

‘If the retirement is like the pandemic; it kind has me thinking... when we’re at home and not being able to go anywhere, I’d say if you were retired and couldn’t go any place, I think I’d crack up’.

(Patrick, Clinical Nurse Manager)

By contrast, nine of the sixteen women nurses say that their experience of working and living through the pandemic has affected their retirement decisions, with most of these saying it has made them want to retire earlier. They cite reasons including concern about the impact of COVID-19 on their own health, about their increased vulnerability to COVID-19 as they age and concern about infecting family members for whom they provide care. For example, one nurse said:

‘And it has made me think... you know, I feel I have worked maybe, very, very hard. And I have worked maybe over and above what I should work. And I, it probably has said to me, look, [Bridget], you have to mind yourself, you have to mind yourself more’.

(Bridget, Clinical Nurse Manager)

One nurse who had experienced a very debilitating long-lasting form of COVID-19 said that this strongly affected her retirement decision. She said her experience of COVID-19 was ‘life-changing’, and she now has a heightened awareness that she may not live to retirement age. Consequently, she now plans to reduce her working hours and to retire earlier than planned:

‘I’m a full-time worker, and I’m even looking at reducing hours now just to see because I sort of feel—you know, you plan for your retirement but you might not get there so... I don’t know if I’ll get back to full-time hours, and... I’ll look into that and even I will be looking to see if I can retire a little bit earlier’.

(Eileen, Clinical Nurse Manager)

She felt that she wasn’t protected as a worker and that now she needs to put her own health and that of her family first. She is very disillusioned with the way nurses were treated during the pandemic.

Others worry that their age puts them at greater risk of contracting COVID-19: another nurse, Marie, stated that prior to the pandemic, she had intended to work until age 65, but now plans to retire up to three years earlier, because of exhaustion and the risk COVID-19 poses to her health, since she is aged over 60:

‘I’m 61, now since March, and it has really been a catalyst for me to look at because of my age, my risk... that life is short, that if I was to pick it [COVID-19] up... And I find that I’m actually very strongly considering retiring. Maybe July 12 months at the latest, you know, even though I had planned to stay to work till I’m 65. But it’s just the same battle, you get exhausted I think, the same fight every day. It just is so challenging’.

(Marie, Assistant Director of Nursing)

She explains that she is in a management role and even though she is somewhat conflicted, she has decided to leave earlier than planned:

‘We have to keep manning this ship and I have to continue being the captain of my department and keep the motivation... So totally, as soon as I can get out and be guaranteed X amount of income, I’m out. And I’m sad, but I have to preserve my own well-being’.

(Marie, Assistant Director of Nursing)

Other women who are caring for/supporting vulnerable family members say that this has been one of the deciding factors in prompting them to take an earlier retirement than initially planned. One woman when asked if her husband’s illness had influenced her to retire earlier responded as follows:

‘Absolutely, absolutely, absolutely. It’s like the last straw, you know, that’s the way you would describe it. I suppose for me, personally, I think. My husband was very sick during the pandemic... which was an added worry for me, because I was so scared of bringing home COVID to him, you know’.

(Valerie, Public Health Nurse)

Another nurse who is caring for and supporting both older relatives and grandchildren outside of work is going to retire earlier than planned. She explains:

‘And I just yeah, I fear for the health effects that are the unknown, I suppose is the bit that I feared the most, and that I’ll bring it home. Em, my husband is older than me, I look after my mother and my mother-in-law. So, I suppose a link in even with more vulnerable than I am, even, at home, you know’.

(Brenda, Public Health Nurse)

Some nurses are conflicted: they want to leave because they are stressed and exhausted, but also simultaneously feel they should stay on to support their colleagues. The following quote from a nurse in a management role illustrates her dilemma and highlights her perception that her age makes working during a pandemic more difficult:

‘I hadn’t thought of retiring, I have to say. But I will... retire in October, and I would have kind of... been happy enough to work... I think it’s a young person’s game... It’s been very hard to... I keep going back to that constant, relentless, you know... I feel I’ve aged ten years in a year... that’s how I feel now. So, I never thought I’d do this. I’m actually counting down the months, okay I have six months to do... I’ll just get through it... But I can see how you can get into that frame of mind... I could go now, if I wanted to. But I won’t, because... I want to leave the team reasonably settled. And... there’s a lot going on in terms of supporting staff and you wouldn’t just like to walk out the door and say, “Cheerio lads”. Not in the middle of a crisis. Like, I just would hate to end work like that’.

(Nora, Assistant Director of Nursing)

Despite her conflicted feelings, she has decided to retire earlier than she originally planned.

### 3.4. Changes in Own Views on Role of Nursing and Public Perception

Nurses were asked whether the pandemic had altered their perspective on the role of nurses. There were a variety of responses, with some saying that it had not changed their view, that nursing remained fundamentally the same. Others say that they believe that the role of nursing has changed, with one nurse stating that both the pandemic and changes in the direction of nursing generally have made it a more supervisory, less tactile occupation.

‘I think it definitely is more of a kind of a more of a supervisory role, I suppose. And... I think it might be just time and the role of the nurse has changed. I don’t know about the pandemic, I think probably just from the tactile point of view—that’s probably where you’re going to see a big difference. It’s not going to be as close contact with patients’.

(Susan, Assistant Director of Nursing)

Conversely, others feel that the pandemic gave them a chance to practice nursing as they felt it should be—offering the opportunity to spend more time with patients and to advocate for them, since they were given support to perform other aspects of their role during the pandemic. Some say the pandemic made them very proud to be nurses, speaking of their adaptability and flexibility and ability to respond in a crisis. One nurse feels that the pandemic has brought out the best in nurses and engendered a sense of solidarity; despite having an underlying health condition, he chose to stay on and show leadership to demonstrate his faith in the health guidelines, giving a good example to his colleagues:

‘But I think this is when we’re at our best. Humanity is at its best when there’s adversity. Like even... I know, there’s been a lot of negativity... And, I just, you know, we’re all in this together. And if you approach it that way, there’s a great sense of coming together’.

(Gerard, Clinical Nurse Manager)

A staff nurse echoes this—she has a sense that it was a one-in-a lifetime opportunity for nurses to be engaged in a crisis, similar to nursing during war time, and to respond well. Some nurses say they now feel more positively than before about nursing and that they have personally become more skilled and confident. Two nurses who were redeployed say the pandemic meant they had gained new skills—one who had previously worked in a community setting said she was now more clinically oriented:

‘So now, I find it’s better for me, it’s actually upped my game, I’m actually doing a post grad in infection prevention control as well... So for me, that has been a huge part of it. So it has upped my clinical skills as well and it’s made me more aware of what I need to do from a nursing perspective, which has been brilliant for me’.

(Catherine, Clinical Nurse Manager)

Discussing the public perception of nursing, some say they were happy that the public applauded healthcare workers during the early months of the pandemic and feel that society has gained a new appreciation of nurses:

‘I think it’s definitely changed the public’s view of the role of nurses... apparently there seems to be record applications to study nursing... People see that no matter what happens in the world, healthcare is essential and vital to all and it’s only when it impacts you, that people realise that it does have serious health implications. And how, that health people, despite the risks to ourselves... just have to keep going into work every day and take care of people. And I think they see that that’s probably a good and a decent thing’.

(Edwina, Clinical Nurse Manager)

However, others feel that this form of appreciation (public applause) will be short-lived and will disappear when the pandemic is over, while others note that this type of public recognition is not matched by appropriate recognition and remuneration from employers. As one nurse puts it:

‘I mean, I know the general public very much do [appreciate nurses]. In a lot of commentary, they do always mention the nurses. But I don’t feel from the point of view of like, you know, some kind of reward or recognition, I think there’s an expectation there that nurses will do this, and nurses will do that. And I think that, that in itself can be very... frustrating, you know, that there isn’t good recognition for the challenges that we have had to face’.

(Bernadette, Assistant Director of Nursing)

Another nurse forcefully expressed the view that public appreciation is only temporary and that nurses’ efforts are taken for granted:

‘And at the beginning... with all that clapping that was going on and all that bullshit like, I would have felt, you know, quite proud of our profession. But now, I kind of see what happens in other professions... I look at the teachers, and... I feel quite resentful of them, actually, because I just feel... they went back to school from September till Christmas, and now they’re gone off the pitch again, until God only knows when, and I feel our profession has just, it’s just been expected. Like we’ve had to put ourselves out there. We’ve lost staff. We’ve... had to put on that damn PPE day in day out, we’ve, you know, we’ve looked after people in spite of it, you know. So if it has changed me, and how I view the profession, I just think society just takes our profession very much for granted. I feel that we’re still basically seen as the handmaidens of the health service. And I wouldn’t have felt that before the pandemic, but I do now, you know’.

(Martina, Assistant Director of Nursing)

Both she and others feel that nurses are not well recognised and rewarded and she is disillusioned with the way nurses are treated:

‘It’s changed my view of work... It definitely has. And as a nurse... you’re just a number. And this might sound very cynical, but in the very beginning everyone’s clapping, saying how great you are, and then... people get back to normal and really... you’re just a nurse... I actually feel so disheartened about nursing now; I would hope my children... don’t go into it because it’s just, it’s awful’.

(Martina, Assistant Director of Nursing)

## 4. Discussion

This article investigates the experiences of older Irish nurses during the COVID-19 pandemic and specifically explores the impacts for their own health and that of their families. It also discusses their views on the public perception of nurses. It provides insights into how their experiences feed into decisions either to retire earlier than planned or to continue working. The latter is important both for building evidence on how pandemics affect retirement decisions for nurses and for informing employer policy for older healthcare workers.

Evidence from our study highlights the impacts of significant under-investment in the Irish public health system over a long period of time, most particularly since austerity policies were introduced following the global financial crisis [12]. This has resulted in pre-existing staff shortages—mentioned by several participants—and this undoubtedly adds to the strain that many Irish nurses feel when they are dealing with the demands of COVID-19.

The staff shortages make it very difficult for Irish nurses to deal with the crisis, particularly since these are exacerbated when colleagues have to isolate. This study provides evidence of inadequate physical facilities for staff (showers, changing rooms) and patients (large wards in some hospitals) that make infection control difficult. It confirms previous findings that nurses are at high risk of personal exposure to the disease, and that some nurses contracted the COVID-19 virus at work, particularly those working in high-risk areas [10]. The likelihood of contracting the virus was compounded by the initial shortage of PPE in Ireland and the guidelines that directed some nurses not to wear masks during the early stages of the pandemic [17,18,19,21]. Nurses made recommendations for infrastructural changes to improve facilities both for nurses and for patients. Some say that the pandemic highlighted the need for staff facilities to be improved, including providing changing and showering facilities and rest rooms [9], and improved ventilation. They also recommended that more beds and more isolation rooms be provided and that large wards where it is very difficult to prevent the spread of infection be replaced with smaller units and that all units in all hospitals be provided with working internet connections, as this is vital for patients to be able to interact with their families.

Redeployment was one of the policies adopted by the HSE to deal with the pandemic. In some cases, this poses problems for nurses; in acute hospital settings, they typically had to move to work in intensive care units in hospitals, or from the community to nursing homes where there was an outbreak of COVID-19, or to testing or vaccinating facilities [19]. While many nurses were willing to be redeployed, their accounts support previous international studies that emphasise difficulties experienced by redeployed nurses in having to upskill very quickly, join a new team and learn new procedures while simultaneously trying to control infection [20]. This suggests a need for ongoing support for redeployed and other nurses, including psychological supports. While there is evidence that the HSE did provide access to counselling, some nurses felt that additional supports such as clinical peer group meetings for debriefing are also needed if they were exposed to large numbers of patients dying, for example, or prolonged stress. There were a number of recommendations to provide additional mental health and well-being supports, similar to calls in recent Irish and international studies [9,39]. Others called for dedicated time to be set aside for staff to become familiar with the myriad of new procedures that need to be followed and for these to be communicated more clearly and concisely [9].

Resonating with international findings, some participants report extreme tiredness and/or burn-out from work stresses, and many are also anxious about possibly transmitting the virus to family members [22,40]. Study participants report experiencing ‘COVID-fatigue’ arising from the duration of the pandemic [27]. At the time of interview, the vaccination programme was being rolled out in Ireland, and participants were either recently vaccinated or hoping to be vaccinated soon, but fearing the impact of new variants. Nurses report that it is difficult to continue working in the face of the third wave of COVID-19, particularly as they often have not had any opportunity to take annual leave. The provision of additional staff to cover absences would help to address this burn-out.

Like their international counterparts, Irish nurses showed great dedication in caring for their patients and were willing to work hard and adopt changes in work practices to care for them during the pandemic [10]. They also displayed great collegiality towards their colleagues and commitment to supporting them [10]. However, this was accompanied for many by a fear of contracting COVID-19 themselves and also of infecting their family members [10]. Some nurses report a perceived unevenness across the HSE in the process by which some nurses with certain conditions were told by their managers to cocoon, while others with similar conditions were not given this option and had to continue working in high-risk areas.

In addition to the negative impacts of the pandemic on their working lives, a small number of nurses reported positive consequences. Some nurses felt it gave them an opportunity to respond positively to a major public health challenge, and this invigorated them and made them feel useful. This resonates with previous international findings [10]. Some participants felt validated by the public applause that was given at the start of the pandemic. Others were less impressed by this, expressing the view that this public approval would be temporary—these varied responses resonate with previous Irish/UK research [32]. Some nurses stated that being given some tangible recognition for their work by the government and employers in the form of increased pay, shorter working hours and/or increased annual leave would be a more meaningful response to their efforts.

A new insight from our findings is that there are notable differences between the perspectives of male and female nurses in relation to the impact of the pandemic on the timing of their retirement. First, a majority of women say they will retire earlier than they had originally planned, while very few men say it has changed their retirement plans. Several men and women said that they have not changed their retirement plans as they need to work until their original date for financial reasons. Those who say they will retire early wish to do so because of the pressures of work, worries about their health and, for some, disillusionment with the way they perceive they have been inadequately protected during the crisis. They say the pandemic has made them realise the importance of prioritising their own health and that of their families. Some of the women nurses provide care for older family members, and it is clear that the desire to avoid infecting their relatives plays a part in their wanting to retire earlier than planned. Nurses recommend that supports need to be provided for nurses who have caring responsibilities for vulnerable family members, including more flexibility to work part time. Some women nurses are very conflicted about whether to stay on at work or retire early—they feel that they should stay to support their colleagues and/or care for their patients, while at the same time they wish to leave earlier for the reasons just outlined. Even though they feel conflicted, most plan to retire earlier than planned.

Due to the fact that our sample consists of 25 older nurses, we do not claim to universalise from our findings to the general population of older nurses in Ireland. However, it does provide insights into the experiences of these nurses and the ways in which it influenced their decision making. It is worth noting that if similar decisions were to be made by other nurses to leave the profession, this may well exacerbate the existing shortage of nurses in Ireland and hinder progress towards extending working lives.

## 5. Conclusions

The main aim of this article is to provide timely insights into the impact of the COVID-19 pandemic for the working lives of older nurses in Ireland, and particularly into whether and how it may affect their retirement timing. The use of a gendered political economy of ageing framework highlights how national and organizational policies, as well as caring norms and responsibilities, affect older nurses experiences and their retirement decisions. The impacts on the work experience of nurses are mainly negative, although with some positive outcomes; this largely supports previous findings in the national and international literature on healthcare workers in general. The article offers new insights into issues that affect older workers specifically. Many of the older women nurses have additional caring responsibilities for older relatives outside work; some consider their age to be an additional risk factor for contracting the virus; both of these factors together are likely to nudge them in the direction of leaving their jobs earlier than previously planned.

The article demonstrates that a combination of factors including stress, burn-out and health impacts for older nurses and their families, particularly in the context of staff shortages, can cause nurses to reappraise their perspective on work, leading them to prioritise their own and their families’ health. One of the original contributions of the paper is the insight it provides into how the pandemic has affected older nurses’ decisions about their retirement timing. This is an issue that is extremely important, firstly, because it provides a valuable contribution to the body of literature on retirement decision making and extending working life and, secondly, because it provides data to inform appropriate policy responses to the challenges raised by the pandemic and to nurse retention more generally. The latter will be particularly relevant in the Irish context of chronic staff shortages and difficulties in retaining nurses.

The following policy recommendations arise from the challenges identified by the research participants. Many nurses felt their work during and outside of the pandemic should be recognised in tangible ways including increased pay, additional annual leave, increased staffing levels and a reduction in working hours. However, at the time of writing, it appears that such measures have not yet been introduced, and unions representing nurses have recently announced that they are taking a case to the Labour Court [41]. Other recommendations include the provision of infrastructure (showers, changing and rest facilities) for staff and for patients (smaller wards, good internet connection). Additional psychological supports for staff, including peer clinical debriefing, would address some of the concerns contributing to the decisions of older workers to leave the workforce prematurely. Frontline staff should be involved in designing policies to address the pandemic. Supports need to be provided to staff who are caring for vulnerable family members—the existing pre-retirement leave scheme should be extended to all staff, not only clinical staff, and where nurses are caring for vulnerable family members, consideration should be given to not requiring them to work in high-risk areas and to providing ready access to more flexible work options for all nurses.

Our findings suggest that there is a need for further international research on the impacts of the pandemic on older nurses—to investigate whether the pandemic has similar impacts in relation to retirement for older nurses in other countries, particularly in view of the identified importance of pre-pandemic staff shortages in Ireland. Such research would ideally use a mixed methods, gendered, life-course approach, which could provide insights into gender differences in nurses’ responses to the impacts of the pandemic across different national and employer contexts.

## Figures and Tables

**Table 1:** Staff grade of participants.

Grade	Staff Nurse/ Senior Staff Nurse/ Public Health Nurse/ Community Mental Health Nurse	Clinical Nurse Manager/ Advanced Nurse Practitioner/ Clinical Nurse Specialist/ Occupational Nurse	Assistant Director of Nursing (ADON)	Total
Female	6	3	7	16
Male	1	6	2	9
Total	7	9	9	25

**Table 2:** Age profile of participants.

Age Group	49–54	55–59	60–64	Total
Male	4	3	2	9
Female	3	8	5	16

**Table 3:** Marital status of participants.

Marital Status	Married/In Long-Term Relationship	Single	Divorced/Separated
Total	16	2	7

**Table 4:** Participants with a caring role outside work.

Caring Role	Male	Female
Total	2	5
